# Predicting the Progression of Chronic Kidney Disease: A Systematic Review of Artificial Intelligence and Machine Learning Approaches

**DOI:** 10.7759/cureus.60145

**Published:** 2024-05-12

**Authors:** Fizza Khalid, Lara Alsadoun, Faria Khilji, Maham Mushtaq, Anthony Eze-odurukwe, Muhammad Muaz Mushtaq, Husnain Ali, Rana Omer Farman, Syed Momin Ali, Rida Fatima, Syed Faqeer Hussain Bokhari

**Affiliations:** 1 Nephrology, Sharif Medical City Hospital, Lahore, PAK; 2 Trauma and Orthopedics, Chelsea and Westminster Hospital, London, GBR; 3 Internal Medicine, Tehsil Headquarter Hospital, Shakargarh, PAK; 4 Internal Medicine, Quaid-e-Azam Medical College, Bahawalpur, PAK; 5 Medicine and Surgery, King Edward Medical University, Lahore, PAK; 6 Surgery, Salford Royal NHS Foundation Trust, Manchester, GBR; 7 Medicine and Surgery, Fatima Jinnah Medical University, Lahore, PAK; 8 Surgery, King Edward Medical University, Lahore, PAK

**Keywords:** systematic review, prediction, disease progression, chronic kidney disease, machine learning, artificial intelligence

## Abstract

Chronic kidney disease (CKD) is a progressive condition characterized by gradual loss of kidney function, necessitating timely monitoring and interventions. This systematic review comprehensively evaluates the application of artificial intelligence (AI) and machine learning (ML) techniques for predicting CKD progression. A rigorous literature search identified 13 relevant studies employing diverse AI/ML algorithms, including logistic regression, support vector machines, random forests, neural networks, and deep learning approaches. These studies primarily aimed to predict CKD progression to end-stage renal disease (ESRD) or the need for renal replacement therapy, with some focusing on diabetic kidney disease progression, proteinuria, or estimated glomerular filtration rate (GFR) decline. The findings highlight the promising predictive performance of AI/ML models, with several achieving high accuracy, sensitivity, specificity, and area under the receiver operating characteristic curve scores. Key factors contributing to enhanced prediction included incorporating longitudinal data, baseline characteristics, and specific biomarkers such as estimated GFR, proteinuria, serum albumin, and hemoglobin levels. Integration of these predictive models with electronic health records and clinical decision support systems offers opportunities for timely risk identification, early interventions, and personalized management strategies. While challenges related to data quality, bias, and ethical considerations exist, the reviewed studies underscore the potential of AI/ML techniques to facilitate early detection, risk stratification, and targeted interventions for CKD patients. Ongoing research, external validation, and careful implementation are crucial to leveraging these advanced analytical approaches in clinical practice, ultimately improving outcomes and reducing the burden of CKD.

## Introduction and background

Chronic kidney disease (CKD) is a progressive and debilitating condition characterized by the gradual loss of kidney function over time, necessitating renal replacement therapy (RRT) such as dialysis or kidney transplantation [1]. The progression of CKD is often insidious, with patients experiencing minimal or no symptoms in the early stages, making early detection and monitoring crucial for preventing further deterioration and potential complications [2,3]. It is a global public health concern with a substantial socioeconomic burden, affecting millions of individuals worldwide. The World Health Organization (WHO) estimates that approximately 10% of the global population is affected by CKD, making it a significant contributor to the global disease burden [4]. Therefore, monitoring and management of CKD progression are essential to delay the onset of end-stage renal disease (ESRD) and reduce the associated morbidity and mortality. Regular assessment of kidney function through blood and urine tests, along with monitoring of risk factors such as hypertension, diabetes, and proteinuria, is vital for tracking disease progression and guiding treatment decisions [5,6].

In recent years, artificial intelligence (AI) and machine learning (ML) techniques have emerged as powerful tools in the field of medicine, offering new avenues for early disease detection, risk prediction, and personalized treatment strategies. In the context of CKD, these advanced analytical approaches hold significant promise in predicting disease progression and identifying high-risk individuals, ultimately facilitating timely interventions and improved patient outcomes [7-9]. By leveraging large volumes of patient data, including demographic information, clinical measurements, laboratory results, and longitudinal records, AI/ML algorithms can uncover intricate patterns and relationships that may not be apparent through traditional statistical methods. These techniques can handle complex, high-dimensional data and capture nonlinear relationships between multiple variables, making them well-suited for predicting CKD progression, which is influenced by a multitude of factors [10,11].

The application of AI/ML techniques in CKD management has several clinical implications. Firstly, accurate prediction models can assist healthcare providers in identifying individuals at high-risk of CKD progression, enabling early interventions and personalized treatment strategies. This proactive approach can potentially slow disease progression and delay the need for RRT, improving patient outcomes and quality of life [12,13]. Secondly, the integration of AI/ML techniques with electronic health records (EHRs) and clinical decision support systems (CDSS) can enhance patient care and resource allocation. By leveraging predictive models, healthcare providers can receive timely alerts or notifications when patients approach critical risk thresholds, facilitating early referrals for nephrology consultations, diagnostic evaluations, and the initiation of appropriate management strategies [14]. Furthermore, AI/ML techniques can aid in the identification of key predictive features and biomarkers associated with CKD progression. This knowledge can guide clinicians in focusing their efforts on monitoring and managing critical risk factors, potentially slowing disease progression and improving patient outcomes. While the implementation of AI/ML techniques in clinical settings presents challenges related to data quality, potential biases, and ethical considerations, ongoing research, and external validation are crucial to ensuring the safe and effective integration of these techniques into clinical practice.

The primary objective of this systematic review is to comprehensively evaluate and synthesize the existing literature on the application of AI and ML techniques for predicting the progression of CKD. Specifically, the review aims to assess the effectiveness and performance of various AI/ML algorithms and models in accurately forecasting the deterioration of kidney function, progression to ESRD, and the need for RRT in individuals with CKD. Additionally, the review seeks to identify the key predictive features, clinical variables, and biomarkers that contribute to the predictive power of these AI/ML models. By critically analyzing and comparing the methodologies, results, and findings across multiple studies, the review endeavors to provide insights into the strengths, limitations, and potential clinical implications of utilizing AI/ML techniques for CKD progression prediction. Ultimately, the systematic review aims to inform future research directions and facilitate the translation of these advanced analytical approaches into clinical practice, with the overarching goal of improving early detection, risk stratification, and personalized management strategies for individuals affected by CKD.

## Review

Materials and methods

Search Strategy

This systematic review adheres to the Preferred Reporting Items for Systematic Reviews and Meta-Analyses (PRISMA) guidelines to ensure transparency and rigor in the review process [15]. We conducted a thorough search of relevant literature across prominent databases, such as PubMed, Embase, Web of Science, and Scopus, renowned for their extensive coverage of medical and scientific literature. These databases were selected for their comprehensive collection of peer-reviewed articles, providing a robust foundation for our systematic review of AI and ML in predicting the progression of CKD. The search strategy employed a meticulously curated set of keywords and phrases aligned with the objectives of the study. These included terms such as "chronic kidney disease," "artificial intelligence," and "machine learning." Boolean operators "AND" and "OR" were strategically utilized to construct the search algorithm. To ensure the inclusion of contemporary and relevant literature, the search was limited to studies in the last five years up to January 2024. This timeframe allowed for the incorporation of the current research, offering a comprehensive overview of AI and ML in predicting the progression of CKD. Filters were applied to include studies published in the English language and those involving human subjects, in line with the objectives of our review. We also conducted manual searches of the reference lists of the included studies and relevant reviews to supplement the electronic database search.

Eligibility Criteria

The eligibility criteria for this systematic review were established to ensure precision and relevance in selecting studies for inclusion. Peer-reviewed research articles, observational studies, clinical trials, and other relevant study designs focusing on AI and ML techniques in predicting the progression of CKD were considered eligible for inclusion, reflecting a commitment to evidence-based knowledge. To maintain methodological rigor, studies published in English language were included, acknowledging English as the predominant language of scientific communication. Conversely, exclusion criteria were carefully tailored to maintain focus and rigor. Studies not directly addressing the prediction of CKD progression using AI and ML techniques, as well as those lacking relevant outcome measures, were excluded. Non-English language publications, unpublished works, and gray literature, such as conference abstracts, were also excluded. Furthermore, studies presenting insufficient data on the prediction of CKD progression using AI and ML techniques were excluded to ensure the integrity of the review's findings.

Data Extraction

The data extraction process was conducted in a meticulous and structured manner to ensure the accuracy and completeness of the review findings. This process involved two stages, emphasizing thoroughness and reliability. In the initial stage, articles were screened based on the relevance indicated by titles and abstracts. Two independent reviewers assessed each article's abstract to determine its relevance to the review's focus. Articles deemed relevant or probably relevant underwent a detailed examination in the second stage. In the second stage, full-text articles meeting the inclusion criteria underwent a detailed data extraction process. Two independent reviewers utilized a standardized data extraction template within Microsoft Excel (Microsoft Corporation, Redmond, Washington, USA) to capture and organize critical information from each study. Any discrepancies between reviewers were resolved through the adjudication of a third independent reviewer.

Results

Study Selection Process

**Figure 1:**
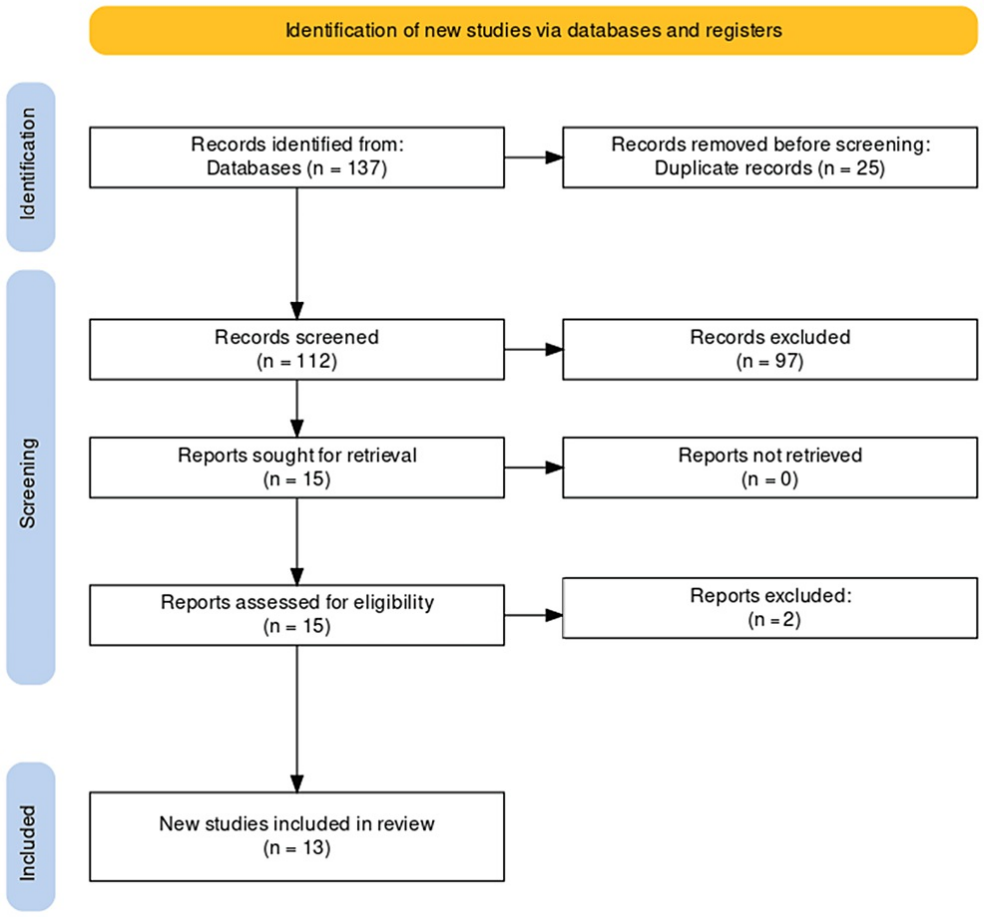
PRISMA diagram showing the selection process of included studies. PRISMA: Preferred Reporting Items for Systematic Reviews and Meta-Analyses.

The study selection process adhered to the PRISMA guidelines, ensuring transparency and systematicity. A comprehensive search initially yielded 137 studies, from which 25 duplicates were removed, resulting in a refined pool of 112 unique studies. Subsequent screening of titles and abstracts led to the exclusion of 97 records that did not meet predefined relevance criteria. A full-text evaluation of the remaining 15 articles resulted in the exclusion of two reports that did not align with stringent inclusion criteria. The culmination of this rigorous selection process identified 13 studies suitable for inclusion in the systematic review, providing a focused and robust source of evidence for the analysis of AI and ML in predicting the progression of CKD. The PRISMA flowchart detailing the study selection process is presented below (Figure 1).

Study Characteristics

The key characteristics of several studies investigating the use of AI and ML techniques for predicting the progression of CKD are shown in Table 1. The studies span a range of years from 2019 to 2023 and originate from various countries, including Japan, China, the Netherlands, Israel, Egypt, and the United States. In terms of sample size, the studies cover a wide range, from relatively small samples of around 500 patients to large-scale analyses involving 550,000 patient records. This variation in sample size reflects the diverse data sources and methodological approaches employed across the studies.

**Table 1:** A detailed summary of studies included in this systematic review. AI: artificial intelligence, ML: machine learning, CKD: chronic kidney disease, NLP: natural language processing, LR: logistic regression, DKD: diabetic kidney disease, SVM: support vector machine, RF: random forest, XGBoost: extreme gradient boosting, FNN: feedforward neural network, k-NN: k-nearest neighbors, SMOTE: synthetic minority over-sampling technique, ESRD: end-stage renal disease, DNN: deep neural network, DT: decision tree, LGBM: light gradient boosting machine, GBDT: gradient boosted decision trees, RRT: renal replacement therapy, CNN: convolutional neural network, BLSTM: bidirectional long short-term memory, GNB: Gaussian Naive Bayes, NB: Naive Bayes, GBT: gradient boosting tree, CHF: congestive heart failure, IHD: ischemic heart disease, LSTM RNN: long short-term memory recurrent neural network, AUROC: area under the receiver operating characteristic curve, AUPRC: area under the precision-recall curve, eGFR: estimated glomerular filtration rate, LDL: low-density lipoprotein, HDL: high-density lipoprotein, SNA: serum sodium, CRP: C-reactive protein, TG: triglycerides, ALB: albumin, Scr: serum creatinine, sAlb: serum albumin, Hb: hemoglobin.

Author	Year	Country	Sample size	AI/ML techniques used	Clinical outcomes	Main findings
Makino et al. [9]	2019	Japan	64,059	NLP, LR, Convolutional Autoencoder, Feature Engineering, Machine Learning Evaluation Techniques and Survival Analysis	Prediction of DKD progression	The constructed predictive model achieved a 71% accuracy in predicting DKD aggravation and demonstrated the significance of longitudinal data in improving prediction performance. Long-term analysis revealed a higher incidence of hemodialysis and cardiovascular events among patients with DKD aggravation, emphasizing the importance of early intervention. These findings highlight the potential of AI-supported predictive models to facilitate timely interventions and reduce adverse outcomes in T2DM patients with DKD.
Xiao et al. [16]	2019	China	551	LR, Elastic Net, lasso regression, ridge regression, SVM, RF, XGBoost, neural network and k-nearest neighbor	Prediction of CKD progression	Linear models, particularly Elastic Net, Lasso Regression, Ridge Regression, and LR, exhibited the highest overall predictive power, with an average AUC and precision exceeding 0.87 and 0.8, respectively. LR achieved the highest AUC of 0.873, accompanied by a sensitivity and specificity of 0.83 and 0.82, respectively. Elastic Net demonstrated the highest sensitivity (0.85), while XGBoost exhibited the highest specificity (0.83). Additionally, the effect size analyses highlighted the importance of certain predictors, notably ALB, Scr, TG, LDL, and eGFR, in the predictability of the models, while other factors like CRP, HDL, and SNA showed lesser significance. The developed online tool can facilitate the prediction of proteinuria progress during follow-up in clinical practice.
Kanda et al. [17]	2019	Japan	7,465	Bayesian networks, SVM	Identifying the progression of CKD	The study found that factors, such as age, gender, hypertension, diabetes, and baseline eGFR significantly influenced CKD progression. Bayesian networks were employed to model these relationships, revealing complex interactions among various factors. SVM models were also utilized to predict CKD progression, achieving promising results in risk assessment. These findings underscore the importance of early detection and management of hypertension and diabetes, particularly in older individuals, to mitigate the risk of CKD progression.
Nagaraj et al. [18]	2020	Netherlands	11789	FNN, k-NN, SMOTE, RF, LR, SVM and elastic-net regularization	Forecasting long-term ESRD risk in patients with type 2 diabetes and nephropathy	The FNN model outperformed other machine-learning algorithms and traditional risk prediction models (such as LR and Cox proportional hazards regression) in terms of accuracy, achieving high AUORC values across three clinical trials (RENAAL, IDNT, and ALTITUDE). This suggests that incorporating multiple baseline demographic and clinical characteristics into a machine-learning model can accurately identify high-risk patients who may benefit from therapy, potentially improving clinical outcomes and patient management in real-world settings.
Segal et al. [19]	2020	Israel	550,000	XGBoost, Word2Vec Algorithm, DNN, Feature Engineering and Selection	Early detection of ESRD	Utilizing data from over 550,000 patient records, the algorithm was trained to predict progression to ESRD in patients with CKD stages 1–4. The model achieved a high predictive performance with a C-statistic of 0.93, sensitivity of 0.715, and specificity of 0.958. Notable features contributing to the model included CHF and IHD as co-morbidities, patient age, and the number of hypertensive crisis events. By employing this algorithm, healthcare providers can receive electronic warning messages when patients are approaching the ESRD risk threshold, facilitating early referral for nephrology consultation, diagnosis, and initiation of management when appropriate. This approach holds promise for improving the timely identification and intervention for patients at risk of developing ESRD, potentially leading to better outcomes and management strategies for CKD patients.
Dovgan et al. [20]	2020	China	8,492	DT, Bagging DT, RF, XGBoost, SVM, Simple Gradient Descendent, Nearest Neighbors, Gaussian Naive Bayes, LR, and NN.	Predict the initiation of RRT among CKD patients	Employing stratified 10-fold cross-validation, the evaluation primarily focused on the AUROC, alongside sensitivity and specificity metrics. Results indicated that LR with time features and data balancing yielded the highest AUROC, emphasizing the effectiveness of time-based features and the importance of data balancing for improving sensitivity and specificity trade-offs. Furthermore, analyses across different prediction periods highlighted the robustness of these findings. While filtering data to include only patients with diabetes did not significantly enhance performance, utilizing all patients for training proved more beneficial, underscoring the importance of larger training datasets over homogeneity.
Krishnamurthy et al. [7]	2021	China	90,000	CNN and BLSTM	Forecast the occurrence of CKD within the next six or 12 months before its onset	Among the models tested, CNN performed the best, with an AUROC curve of 0.957 for six-month predictions and 0.954 for 12-month predictions. The study identified key predictors of CKD, including diabetes mellitus, age, gout, and medications such as sulfonamides and angiotensins. These findings suggest that the developed machine learning model could serve as a valuable tool for policymakers in predicting CKD trends, enabling early detection, resource allocation, and patient-centric management strategies.
Su et al. [8]	2022	China	858	LR, RF, XGBoost, SVM, and GNB	Prediction of CKD progression to ESRD	The RF classifier with SMOTE showed the best predictive performance, particularly for patients with early- and advanced-stage CKD. Key features for prediction differed between the early and advanced stages, with urine creatinine and serum creatinine being important predictors, respectively. The RF classifier demonstrated optimal performance, achieving high AUROC values for predicting progression within specified time frames. This study suggests that ML models, particularly the RF classifier, can accurately predict CKD progression, aiding in early diagnosis and management to prevent kidney failure.
Abdel-Fattah et al. [21]	2022	Egypt	400	DT, LR, NB, RF, SVM, and GBT Classifier	Prediction of CKD	SVM, DT, and GBT Classifier with selected features achieved the best performance, reaching 100% accuracy. Relief-F feature selection method demonstrated superior performance compared to chi-squared feature selection and using full features. Additionally, the study optimized model parameters through grid search with cross-validation and evaluated outcomes using metrics such as accuracy, precision, recall, and F1-measure. These findings underscore the potential of hybrid machine learning techniques integrated with feature selection methods for accurate prediction of CKD, offering valuable insights for early detection and treatment of the disease.
Lee et al. [22]	2022	China	11,661	RF, XGBoost, LGBM, GBDT and extra trees	Risk prediction of ESRD in sepsis survivors with CKD	A total of 11,661 sepsis survivors were identified from a database of CKD patients, and during a median follow-up of 3.5 years, 11.7% of them developed ESRD after hospital discharge. ML algorithms were used to predict ESRD risk, with the GBDT model showing the highest performance, achieving an AUROC of 0.879. The model identified estimated glomerular filtration rate (eGFR) <25 mL/min/1.73 m^2^ at discharge, hemoglobin <10 g/dL, and urine protein/creatinine ratio >2 mg/mg as crucial predictors of ESRD. The model outperformed conventional risk scoring systems, indicating its potential utility in identifying sepsis survivors at high-risk of ESRD development. External validation is needed to assess its generalizability.
Zou et al. [10]	2022	China	390	Gradient boosting machine, SVM, LR, and RF	Risk prediction model of ESRD in T2DM and DKD	Using ML algorithms and data from 390 Chinese patients with T2DM and DKD confirmed by renal biopsy, the researchers identified five major predictive factors for ESRD: Cystatin C, serum albumin (sAlb), hemoglobin (Hb), 24-hour urine urinary total protein, and estimated glomerular filtration rate (eGFR). The RF algorithm exhibited the highest performance in predicting ESRD, with an AUROC of 0.90 and accuracy of 82.65%. These findings highlight the importance of assessing kidney function, nutrition, and anemia in patients with T2DM and advanced DKD to delay the progression to ESRD, rather than focusing solely on age, sex, and control of hypertension and glycemia as previously emphasized. The developed nomogram based on these factors provides a practical tool for clinicians to predict individual renal survival probability and guide early intervention strategies.
Inaguma et al. [23]	2022	Japan	5,657	LR and RF	Prediction model for the extremely rapid decline in estimated GFR in CKD	The study applied a unique clustering approach to classify patients into nine trajectory clusters based on eGFR decline rates and baseline eGFR levels. The RF algorithm was utilized to develop prediction models for each cluster, achieving AUROC values of 0.69, 0.71, and 0.79 for high, intermediate, and low baseline eGFR groups, respectively. Important features identified for prediction included hemoglobin, albumin, and C-reactive protein levels. However, limitations such as biases in electronic health record data and differences in calibration among patient groups were acknowledged, indicating the need for further validation and prospective studies to refine prediction models.
Zhu et al. [11]	2023	USA	82,667	LSTM RNN, Feature Selection Analysis, Cox Proportional Hazards Models, RF and LightGBM	Prediction of CKD progression using recurrent neural network	LSTM RNN consistently achieves high prediction accuracy with average AUROC exceeding 0.95 and AUPRC exceeding 0.83. LSTM RNN outperforms baseline methods across all scenarios, except for predicting disease progression within 90 days where the dynamic Cox proportional hazards model achieves similar AUPRC. Using all variables yields better prediction performance for LSTM RNN and Cox models, particularly for the 365-day prediction window. Moreover, LSTM RNN performance improves with larger prediction windows. Variable selection analysis identifies longitudinal estimated eGFR as the most predictive variable, with additional variables marginally improving prediction accuracy. The study underscores LSTM RNN's superiority in utilizing longitudinal EHRs for CKD progression prediction, offering advantages in accuracy and simplicity compared to existing methods.

The AI/ML techniques utilized in these studies encompass a broad spectrum of algorithms and methods, including natural language processing (NLP), logistic regression (LR), elastic net, lasso regression, ridge regression, support vector machines (SVM), random forests (RF), XGBoost, neural networks, k-nearest neighbors (k-NN), Bayesian networks, convolutional neural networks (CNN), bidirectional long short-term memory (BLSTM), feature engineering, decision trees (DT), gradient boosting machines, and long short-term memory recurrent neural networks (LSTM RNN). The clinical outcomes investigated in these studies primarily focus on predicting the progression of CKD to end-stage renal disease (ESRD) or the initiation of renal replacement therapy (RRT). Some studies also aim to predict the aggravation or progression of diabetic kidney disease (DKD), proteinuria, or the rapid decline in estimated glomerular filtration rate (eGFR) [9,10,18,23]. In terms of main findings, the studies generally report promising results in utilizing AI/ML techniques for predicting CKD progression, with many models achieving high accuracy, sensitivity, specificity, and area under the receiver operating characteristic (AUROC) curve scores. Several studies highlight the importance of incorporating longitudinal data, baseline characteristics, and specific biomarkers or clinical features in improving prediction performance [7,10,16,23].

Discussion

AI and ML techniques have emerged as powerful tools in the field of healthcare, offering new avenues for early disease detection, risk prediction, and personalized treatment strategies. In the context of CKD, these advanced analytical approaches hold significant promise in predicting disease progression and identifying high-risk individuals, ultimately facilitating timely interventions and improved patient outcomes [12,24,25]. The studies summarized in Table 1 highlight the potential of AI/ML techniques in predicting the progression of CKD to ESRD or the need for RRT. By leveraging large volumes of patient data, including demographic information, clinical measurements, laboratory results, and longitudinal records, these algorithms can uncover intricate patterns and relationships that may not be apparent through traditional statistical methods. One of the key advantages of AI/ML techniques is their ability to handle complex, high-dimensional data and capture nonlinear relationships between multiple variables. This capacity is particularly valuable in the context of CKD, where disease progression is influenced by a multitude of factors, such as age, comorbidities, genetic predisposition, and environmental exposures. Traditional risk prediction models often struggle to account for these intricate interactions, leading to suboptimal performance.

The included studies employed a diverse range of AI/ML algorithms, including logistic regression, support vector machines, random forests, gradient boosting, neural networks, and deep learning approaches like CNNs and LSTM recurrent neural networks. Each of these techniques offers unique strengths and capabilities, and their performance may vary depending on the specific characteristics of the dataset and the prediction task at hand. Notably, several studies reported promising results, with models achieving high accuracy, sensitivity, specificity, and AUROC curve scores in predicting CKD progression. For instance, Segal et al. (2020) developed an algorithm that achieved a C-statistic of 0.93, sensitivity of 0.715, and specificity of 0.958 in predicting ESRD progression in patients with CKD stages 1-4 [19]. Similarly, Krishnamurthy et al. (2021) employed a CNN model that demonstrated an AUROC curve of 0.957 for predicting CKD occurrence within the next six months and 0.954 for 12-month predictions [7]. These findings underscore the potential of AI/ML techniques to serve as valuable decision-support tools for healthcare professionals, enabling early identification of individuals at high-risk of CKD progression and facilitating timely interventions and personalized treatment strategies.

Furthermore, several studies highlighted the importance of incorporating longitudinal data and specific clinical features or biomarkers in enhancing prediction accuracy. For instance, Makino et al. (2019) demonstrated the significance of longitudinal data in improving prediction performance for DKD progression [9]. Similarly, Zhu et al. (2023) reported that including longitudinal eGFR measurements significantly improved the prediction accuracy of their LSTM RNN model for CKD progression [11]. The identification of key predictive features and biomarkers is another crucial aspect of these studies. Various factors, such as age, gender, comorbidities (e.g., hypertension, diabetes), baseline eGFR, proteinuria, serum albumin, hemoglobin levels, and inflammatory markers like C-reactive protein, were found to be significant predictors of CKD progression across multiple studies [10,16,17,23]. This knowledge can guide clinicians in focusing their efforts on monitoring and managing these critical risk factors, potentially slowing disease progression and delaying the need for RRT.

Additionally, the integration of AI/ML techniques with EHRs and CDSS holds immense potential for improving patient care and resource allocation [7,11]. By leveraging these predictive models, healthcare providers can receive timely alerts or notifications when patients approach critical risk thresholds, enabling early referrals for nephrology consultations, diagnostic evaluations, and initiation of appropriate management strategies. However, it is important to acknowledge the limitations and challenges associated with the implementation of AI/ML techniques in clinical settings. Data quality, potential biases, and ethical considerations related to data privacy and transparency must be carefully addressed. Additionally, external validation and prospective studies are necessary to assess the generalizability and real-world performance of these predictive models across diverse patient populations and healthcare systems.

## Conclusions

Artificial intelligence and machine learning techniques hold promising potential in predicting the progression of CKD and identifying high-risk individuals. By leveraging the power of advanced analytical approaches and integrating them with clinical decision support systems and electronic health records, healthcare providers can potentially improve early disease detection, facilitate timely interventions, and optimize patient management strategies for CKD. Nevertheless, ongoing research, external validation, and careful consideration of ethical and practical implications are crucial to ensure the safe and effective implementation of these techniques in clinical practice, reduce the burden of CKD, and ultimately improve outcomes for individuals affected by this debilitating condition.
